# NO SILVER BULLET: ADDRESSING COGNITIVE HEALTH IN RACIALLY AND ETHNICALLY DIVERSE OLDER ADULTS

**DOI:** 10.1093/geroni/igac059.1109

**Published:** 2022-12-20

**Authors:** Elizabeth Muñoz

**Affiliations:** The University of Texas at Austin, Austin, Texas, United States

## Abstract

The growing racial and ethnic diversity of the older adult population, along with continued inequities in brain health outcomes highlight the need to examine risk-factors for reduced cognitive health in racial-ethnic minoritized adults. Stress is an important risk factor for reduced cognitive health, but the conditions under which stress operates among minoritized older adults is poorly understood. This presentation will discuss how studying sources of stress across multiple levels of analyses, including neighborhoods and interpersonal interactions, and the timing and duration of these experiences is needed. Further, addressing within group heterogeneity and shifting from between group comparison will enrich our understanding of risk-factors. Theoretical and empirical support for these propositions will be discussed, particularly as they relate to how neighborhood contexts and experiences with discrimination are associated with reduced cognitive health across multiple timescales.

